# Spatio-temporal spread and evolution of Lassa virus in West Africa

**DOI:** 10.1186/s12879-024-09200-8

**Published:** 2024-03-14

**Authors:** Xia Wang, Xianwei Ye, Ruihua Li, Xiaodong Zai, Mingda Hu, Shaoyan Wang, Hongguang Ren, Yuan Jin, Junjie Xu, Junjie Yue

**Affiliations:** 1 Laboratory of Advanced Biotechnology & State Key Laboratory of Pathogen and Biosecurity, Beijing Institute of Biotechnology, Beijing, 100071 China; 2 Medical College of Guizhou University, Guiyang, 550025 China; 3 Guizhou Provincial People’s Hospital, Guiyang, 550002 China

**Keywords:** Lassa virus, Phylogeography, West Africa, Transmission, Variation

## Abstract

**Background:**

Lassa fever is a hemorrhagic disease caused by Lassa virus (LASV), which has been classified by the World Health Organization as one of the top infectious diseases requiring prioritized research. Previous studies have provided insights into the classification and geographic characteristics of LASV lineages. However, the factor of the distribution and evolution characteristics and phylodynamics of the virus was still limited.

**Methods:**

To enhance comprehensive understanding of LASV, we employed phylogenetic analysis, reassortment and recombination detection, and variation evaluation utilizing publicly available viral genome sequences.

**Results:**

The results showed the estimated the root of time of the most recent common ancestor (TMRCA) for large (L) segment was approximately 634 (95% HPD: [385879]), whereas the TMRCA for small (S) segment was around 1224 (95% HPD: [10301401]). LASV primarily spread from east to west in West Africa through two routes, and in route 2, the virus independently spread to surrounding countries through Liberia, resulting in a wider spread of LASV. From 1969 to 2018, the effective population size experienced two significant increased, indicating the enhanced genetic diversity of LASV. We also found the evolution rate of L segment was faster than S segment, further results showed zinc-binding protein had the fastest evolution rate. Reassortment events were detected in multiple lineages including sub-lineage IIg, while recombination events were observed within lineage V. Significant amino acid changes in the glycoprotein precursor of LASV were identified, demonstrating sequence diversity among lineages in LASV.

**Conclusion:**

This study comprehensively elucidated the transmission and evolution of LASV in West Africa, providing detailed insights into reassortment events, recombination events, and amino acid variations.

**Supplementary Information:**

The online version contains supplementary material available at 10.1186/s12879-024-09200-8.

## Introduction

Lassa fever (LF) is an acute viral hemorrhagic disease caused by Lassa virus (LASV) [1], with a case fatality rate of approximately 30% in hospitals in Nigeria [2]. LF is widespread in West Africa, particularly in Nigeria and the Mano River Union (MRU), which includes Guinea, Liberia, and Sierra Leone. In the past two decades, cases have also been reported in Mali, Côte d'Ivoire, Ghana, Benin, and Togo [3]. LASV is a single- stranded RNA virus with the genome consisting of two segments, the large (L) segment encoding RNA-dependent RNA polymerase (LP) and zinc-binding protein (ZP), and the small (S) segment encoding the glycoprotein precursor (GPC) and nucleoprotein (NP) [4]. LASV belongs to the genus *Mammarenavirus* and the family *Arenaviridae*, with its reservoir is a rodent of the genus *Mastomy* known as “multimammate rat”. And LASV is transmitted to humans mainly through food or household items contaminated by infected rats’ urine and faeces.

Early studies identified at least seven lineages: lineages I to III were circulating in Nigeria, while lineage IV was found in MRU [5]. The strains isolated from Mali and Côte d'Ivoire were proposed as lineage V [6]. The new strain (*Hylomycus pamfi*) isolated from Nigeria, as well as the strains from nosocomial outbreaks in Togo and Benin, may be considered lineages VI and VII [7–9]. Subsequent studies further refined the sub-lineages within each lineage [10, 11]. While the origin of LASV had been reported [12], comprehensive information regarding its spread in West Africa and the phylodynamic characteristics is still limited.

Different strains could exchange genetic information through intergenic reassortment and recombination, leading to the generation of new genotypes and phenotypes, thereby driving viral evolution [13]. LASV belongs to the *Arenaviridae* family, where reassortment and recombination events had been detected, indicating their role in the evolutionary process within this viral family [14, 15]. Although some studies had mentioned the reassortment strains of LASV [11, 12], a global description of recombination events was lacking.

As the sole antigen on the viral surface, GPC was commonly used as an immunogen in vaccine development. The GPC virion forms a trimer, consisting of the receptor-binding subunit GP1 and the transmembrane fusion-mediating subunit GP2. It also contains a stable signal peptide (SSP), which remains as part of the complex in the virus particles. Additionally, GPC contained 11 N-glycosylation motifs, which combine with nearby amino acids to form glycans that affect the immune response [16]. While the study mentioned the variation range of GPC among lineages [17], the specific substitutions were not described in detail.

Based on the above information, we comprehensively reconstructed the spatio-temporal and phylodynamics of LASV in West Africa using as many sequences as possible from public databases, and detected possible reassortment and recombination events in sequences. Finally, we compared the variations of GPC among lineages to gain a global understanding of LASV.

## Materials and methods

### Data preparation and alignments

All sequences up to 2018 were downloaded from the National Center for Biotechnology Information (NCBI), resulting in 1322 initial genome sequences. After removing sequences with lengths less than 95% and incomplete strain attribute information (collection time and location), a set of 500 genome sequences was finally obtained (Additional file 1). These nucleotide sequences were aligned as amino acids using MAFFT v7.490 [18]. Subsequently, the aligned amino acid sequences were converted back to nucleotide sequences to maintain codon alignment.

IQ-TREE v2.0.3 [19] was utilized to construct maximum likelihood (ML) trees for the genomic sequences. A bootstrap value of 1000 was employed to assess the reliability of the ML tree and obtain the best possible score.

### Phylogeographic reconstruction

To reduce computational complexity during phylogeographic reconstruction, cd-hit v 4.8.1 [20] with a threshold of 0.98 was used to select representative strains. The goal was to ensure that there was at least one strain in each branch of the ML trees, as well as in each time, location, and host category. This resulted in 151 L segment sequences that met these criteria, and their corresponding S segment sequences were selected as well.

Phylogeographic inferences were performed using BEAST v1.10.4 [21] with the BEAGLE library [22] to improve computational efficiency. The substitution processes were modelled according to a HKY parametrization [23], and Bayesian skyline plot (BSP) coalescent model was specified as tree topology prior [24]. Relaxed clock models with rates drawn from an underlying lognormal distribution [25] were applied to both segments. Markov chain Monte Carlo chains were run for more than 300 and 200 million iterations for segments L and S, respectively. The chains were sampled every 20,000 generations, and other parameters used default values. Convergence and mixing properties were inspected using Tracer v1.7.2 [26] to ensure that estimated sampling size values associated with estimated parameters were all great than 200. Maximum clade credibility (MCC) trees were summarized using TreeAnnotator v1.10.4 [21] from 10,000 trees regularly sampled from each posterior distribution. The resulting trees were visualized using Figtree v1.10.4 [21], and the dynamic process was visualized using SPREAD3 [27].

### Reassortment and recombination detection

To detect recombination events in the genomic sequences, RDP, GENECONV, BOOTSCAN, CHIMAERA, and MAXCHI of RDP5 [28] were employed. Only recombination events longer than 300 bp and detected by at least three of the five methods with a *P*-value cutoff of 0.05 were considered significant [5]. SimPlot [29] was subsequently used to describe inter-lineage recombination and identify breakpoints with their parent strains.

If a breakpoint was detected at the junction of two segments in the genomic sequences, it would be considered a reassortment event. Further ML trees were constructed to determine whether the topological structure of the strains in the two segments was consistent, and the tanglegram analysis was performed by “matplotlib” in Python.

### Variation feature evaluation

GPC amino acid sequences were retrieved from NCBI. Sequences with a length of less than 95% of the full length were excluded, resulting in a final set of 621 GPC amino acid sequences. The Pinneo strain was selected as the reference sequence for comparison with other strains to assess variation features in GPC. The Shannon entropy of GPC was calculated using a Python script written by Joe R. J. Healey (https://github.com/jrjhealey/bioinfo-tools/blob/master/Shannon.py). Then, the LASV GPC structure file was downloaded from the RCSB Protein Data Bank.

## Results

### Reconstruction of the spatio-temporal dynamics of LASV propagation

**Fig. 1:**
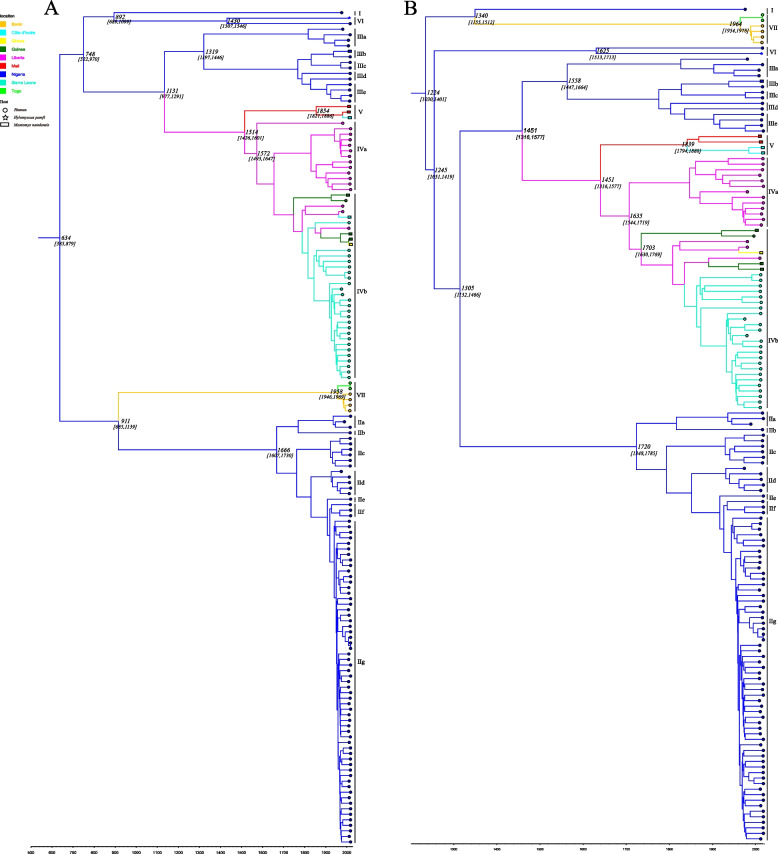
BEAST analysis of complete or almost complete L and S segment nucleotide sequences. The resulting trees for the L segment (**A**) and S segment (**B**) were visualized using Figtree v1.10.4. The node ages, in years, are included on the major nodes, with the 95% confidence ranges displayed in parentheses below the median node ages. The isolates are grouped by their lineages, as represented by the bars to the right of the trees. The reverse axis represents the age, in years, from the most recent isolate. Colours indicate different geographical origins

The time-scaled phylogeographic MCC trees of 151 LASV sequences and the root state posterior probability are illustrated in Fig. 1. Both LASV segments originated in Nigeria, and further results showed that the estimated the time of the most recent common ancestor (TMRCA) for the root of the L segment was approximately 634 (95% HPD: [385879]), whereas the TMRCA of the S segment was around 1224 (95% HPD: [10301401]), other nodes are shown in Table S1. Compared to other study [12], we had more comprehensively considered representative strains from seven lineages to estimate the older root TMRCA of LASV. Overall, the results still showed that the root TMRCA of L segment was earlier than the S segment.

**Fig. 2:**
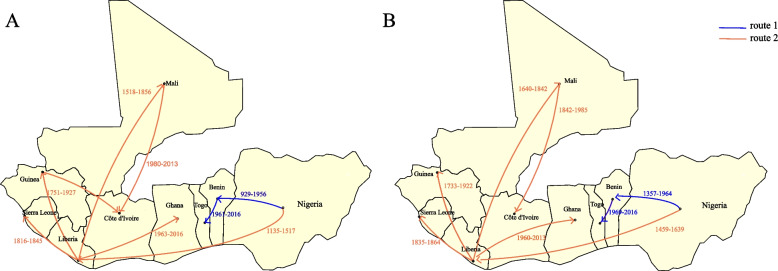
Temporal dynamics of LASV geographic dispersal in West Africa. The plot simply shows the geographic diffusion model of the L segment (**A**) and S segment (**B**) of the LASV over time. The blue line represents route 1, and the orange line indicates route 2. The number displays the time of diffusion from country 1 to country, and they are all within the 95% confidence interval of TMRCA

To further our understanding of LASV, we estimated the geographic diffusion characteristics of LASV in West Africa based on MCC trees. The result indicated that LASV had two main transmission routes in West Africa (Fig. 2). Route 1 originated from Nigeria, passed through Benin, and ultimately reached Togo. While route 2 also started from Nigeria, spread westward to Liberia, and then spreads through Liberia to Guinea, Sierra Leone, Mali, Côte d'Ivoire and Ghana. The transmission patterns of the two segments in LASV were similar, but the transmission time before each location of the S segment was later than that of the L segment. This may be related to the fact that the root TMRCA of the S segment is later than that of the L segment, which also suggests that they may be facilitated by the transmission of two independent lineages [30, 31]. Additionally, we discovered that the strains from Côte d'Ivoire had distinct origins for the S segment and the L segment. The Côte d'Ivoire strains in the L segment were connected to the strains from Mali and Guinea, whereas in the S segment, they were solely linked to the strains from Mali. This suggested that during the ongoing transmission process, the imported L segment sequences in Côte d'Ivoire may have been a result of recombination involving strains from Mali and Guinea.

### Estimation of the phylogeographic features of LASV

**Fig. 3:**
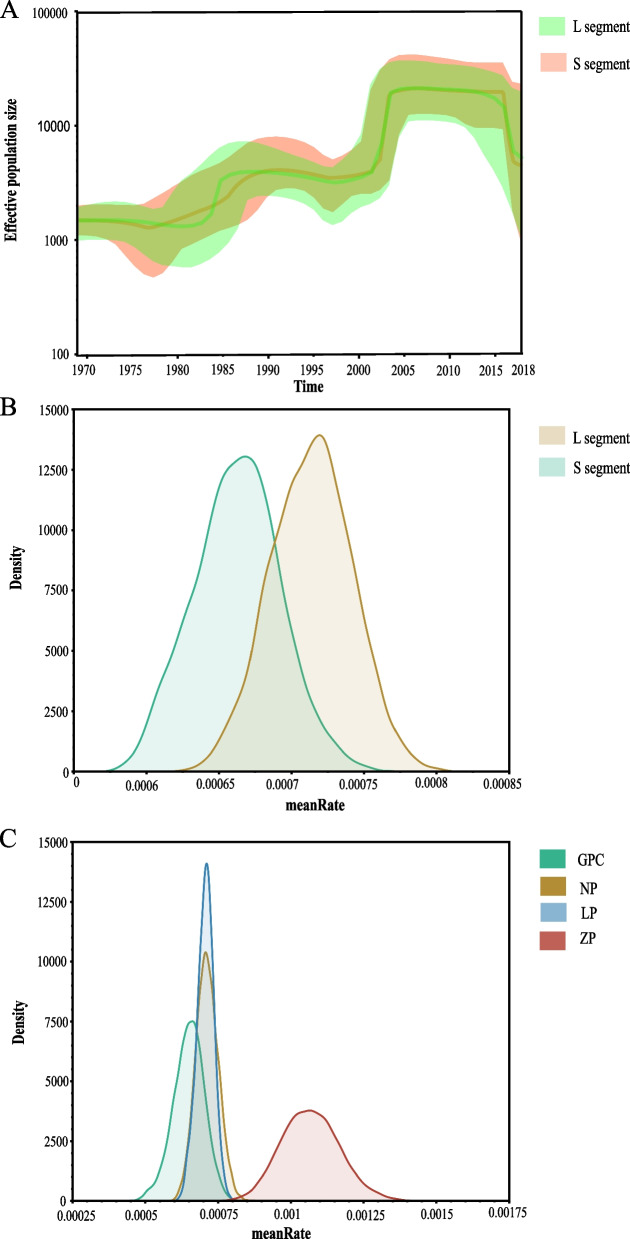
Phylogenetic features of LASV in West Africa. **A** Effective population size (genetic diversity) of the spatial temporal reconstruction of LASV. The thick solid line indicates the median value, and the dark area is the 95% HPD of the genetic diversity estimates. The L segment and S segment are represented in green and light red, respectively. **B-C** meanRate estimation of LASV. **B** Grass green represents the S segment (5.366E-4), and the other segment is light yellow (5.9831E-4); **C** GPC (4.9877E-4), NP (5.4523E-4), LP (5.9939E-4) and ZP (9.857E-4) are expressed in light green, light yellow, light blue and light red, respectively. The horizontal axis indicates the estimated mean rate, and the vertical axis represents the corresponding population density

To investigate the genetic diversity changes during the migration of LASV in West Africa, we employed BSP to reconstruct its demographic history. Figure 3A showed the BSP reconstruction of the L and S segments in which temporal changes of effective population size was plotted. For the L segment, the effective population size underwent a nearly stable period from 1970 to 1975. From approximately 1975, the first rapid increase in genetic diversity started, and around from 1985 to 2000, the effective population size stabilized again. Then, starting from 2000, the second rapid growth occurred and reached its maximum level around 2005, lasting for more than 10 years. Although there has been a decline since 2015, the population size during this period remained higher than during the first stage. Analysis of the BSP for S segment showed that except for slight differences in the first rapid increase period, the trends and time of other demographic patterns were similar.

Due to the observation of inconsistent root TMRCA between the two segments of LASV, we calculated the evolutionary meanRate of two segments and found the evolutionary meanRate of the L segment was faster than that of the S segment (Fig. 3B). Further analysis of the proteins they encoded revealed that ZP had the fastest evolution meanRate, with the rate almost 1.5 times that of other encoded proteins (Fig. 3C). This was consistent with the result described by O. K. Oloniniyi [32], which documented the fastest evolution rate of ZP within lineage II.

### Reassortment and recombination affect the lineage expansion of LASV

Previous reports had indicated that recombination events may lead to strains exhibiting different evolutionary branches in phylogenetic tree [15], and our MCC trees also revealed variations in the topological structures of LASV strains in the two segments. To investigate the underlying reasons for this, we conducted relevant detections, and the specific results were presented in Table 1.

**Table 1:** Detected reassortment and recombination strains by RDP5

Type	Strain	Major parent	Minor parent	*P* value				
				**RDP**	**GENECONV**	**Bootscan**	**Maxchi**	**Chimaera**
reassortment	LASV_3625_V	BA366_ IVb	LASV_3604_V	8.16E-213	2.37E-167	8.95E-201	2.74E-58	2.86E-54
	LASV_3523_V	Mad39_ IVb	LASV_3604_V	3.27E-190	1.05E-139	NS	8.71E-59	4.96E-53
	LASV_3713_V	G1529_ IVb	Mad63_ IVb	3.32E-160	2.09E-105	5.48E-170	2.45E-54	1.30E-22
	LASV_3715_V	G2431_ IVb	Mad69_ IVb	6.42E-156	4.59E-108	1.63E-179	8.40E-56	7.49E-23
	LASV250_IIg [12]	IRR-006_IIg	LASV1015_IIg	2.21E-50	5.84E-46	2.51E-53	1.68E-21	5.89E-19
	IRR-016_IIg	LASV0070_IIg	ISTH1096_IIg	2.49E-36	5.24E-04	1.87E-23	3.48E-24	2.76E-19
	LASV0159_IIg	ISTH-0664_IIg	LASV0104_IIg	2.80E-07	4.06E-02	6.81E-05	4.37E-06	1.08E-06
	LASV003_IIg	LASV241_IIg	LASV242_IIg	1.19E-06	NS	NS	1.52E-05	6.33E-06
	LASV263_IIg [12]	LASV975_IIg	IRR-017_IIg	7.64E-05	NS	NS	1.72E-04	1.44E-02
recombination	LASV_3625_V	BA366_ IVb	LASV_3604_V	9.25E-71	8.66E-57	4.44E-65	1.94E-12	3.69E-11
	LASV_3713_V	Mad63_ IVb	Bamba-R114_V	2.93E-57	1.44E-26	1.96E-54	8.56E-12	3.60E-09
	LASV_3715_V	Mad69_ IVb	Bamba-R114_V	5.20E-54	6.03E-21	6.17E-43	2.46E-12	5.78E-09
	LASV_3523_V	Mad39_V	LASV_3604_V	1.23E-50	1.14E-28	1.15E-48	2.51E-09	3.27E-07

*NS* No significant, *P* value was recorded for this recombination event using this method

**Fig. 4:**
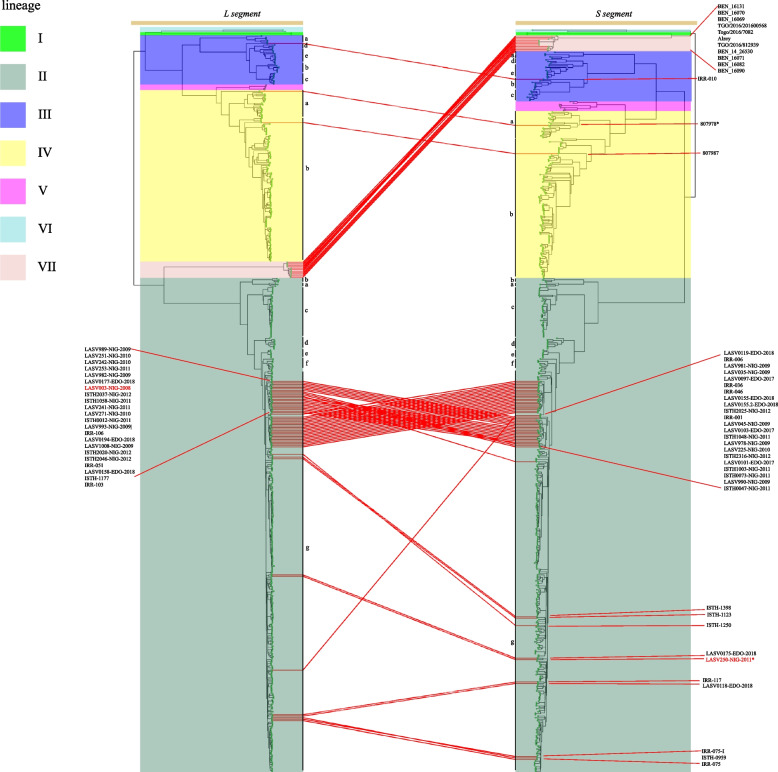
Tanglegram between phylogenetic trees of LASV segments. Phylogenetic trees of the L segment and S segment were constructed using ML. each colour represented one lineage, and the green dots at the end of the tree was a strain. And those strains with different topological structures in the two segments were connected by red lines

In the whole genome, nine strains showed significant reassortment events. The first four strains all belong to lineage V, and their major and minor parents mainly came from lineage IV and lineage V. As for the remaining strains, they all came from the sub-lineage IIg, and the detected results also showed that their parent strains also isolated from the same sub-lineage. And the strains LASV250 and LASV263 had been proven to be reassortment strains [12], indicating that there was reassortment phenomena within the sub-lineage IIg. Figure 4 further demonstrated that in addition to these strains, there may also be some strains that had undergone reassortment, especially the LASV003 strain, which showed strong reassortment signals in two methods. Additionally, it was worth noting that the strain of lineage VII did not show any reassortment signals in detection by RDP5, but in Fig. 4, it clearly showed its distinct topological difference in the two segments, which may also be related to potential reassortment effects [33].

**Fig. 5:**
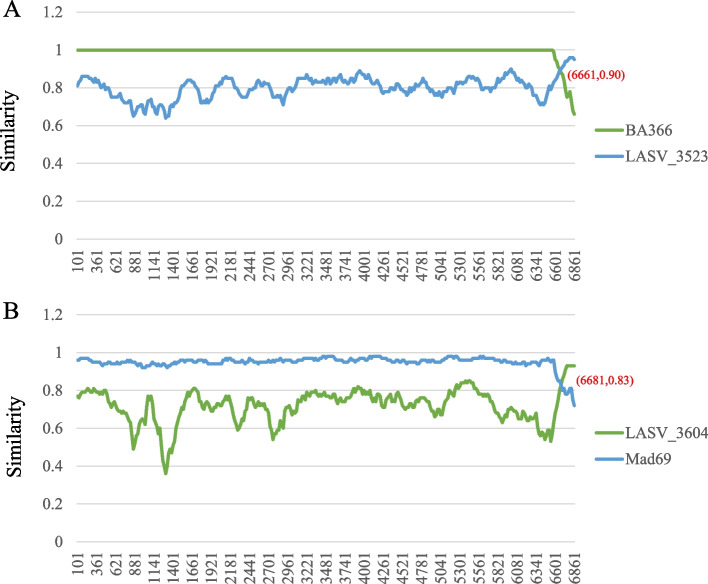
Similarity between recombined strains and their parents. The strains LASV_3625 (**A**) and LASV_3523 (**B**) show their own similarity with their parents. The major parent and minor parent are represented by the green line and blue line, respectively. The red coordinate points represent the breakpoint position and the similarity

Then, we detected the occurrence of recombination within the segment. The results showed that no recombination signals were detected in the S segment, while in the L segment, four strains isolated from Côte d'Ivoire showed significant recombination signals (Table 1). The LASV_3625 strain belonged to lineage V, and its major parent (BA366) came from lineage IV, while its minor parent (LASV_ 3604) was from lineage V. Figure 5A showed before the recombination breakpoint (6661), the similarity between this strain and BA366 reached almost 100%, while after the breakpoint, the similarity with LASV3604 was higher. LASV_ 3713 and LASV_ 3715 was similar to the LASV_3625, with both parents from lineage IV and lineage V, and the similarity before breakpoint remained around 100% with the major parent (Figure S2A). The last strain (LASV_3523) was different from the above strains, its major parent (Mad39) and minor parent (LASV_3604) were isolated from lineage V, and before the breakpoint, the similarity with the major parent was around 90% (Figure S2B). The four detected strains all isolated from lineage V, and their respective parental results showed that some strains of lineage IV and lineage V recombined, leading to the formation of most strains in Côte d'Ivoire and further exacerbating the expansion of lineage V.

Previous study showed the intragenic recombination could play a role in driving the evolution of virus [34]. However, it was unclear whether intragenic recombination could contribute to gene mutations in LASV. Here, we conducted recombination detection on four encoding proteins, but no recombination signals were detected, which indicating that intragenic recombination did not contribute to the evolution of LASV.

### GPC variations among lineages showed the diversity and antigen change of LASV

**Fig. 6:**
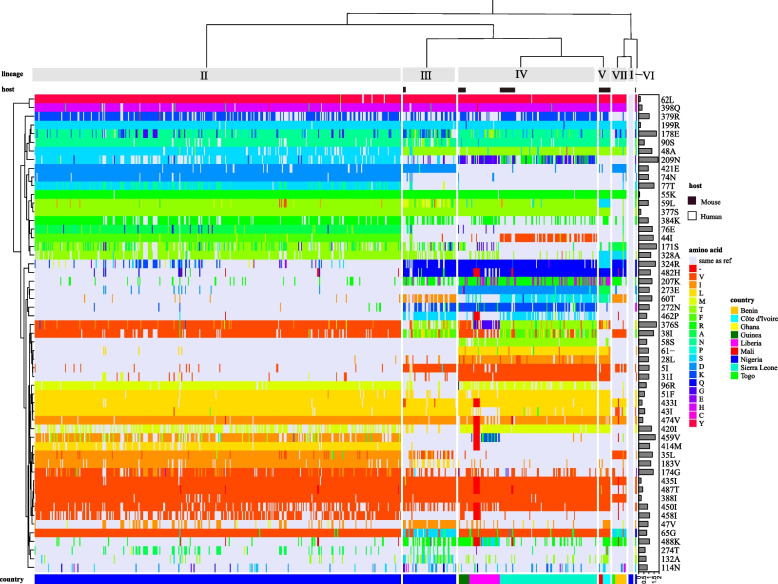
GPC variation characteristics of LASV. The amino acid variation of GPC in different lineages is shown. Different colours on the clustering tree represent different amino acids, and the “lavender” represents the same amino acid as the reference strain. The colours outside the cluster tree represent different countries, hosts and lineages accordingly. The bar graph on the right shows Shannon entropy at these sites

GPC was the main surface membrane protein of LASV, and it mediated the virus fusion [35]. Research had shown that inter-lineage variations in the GPC ranging from 4.9% to 11.0% [17]. To understand these changes, we compared the sequences of 621 GPC and identified 55 amino acids with significant changes (Fig. 6, Additional file 2).

The substitution of isoleucine with valine occurred in I5, I31, I38, I43 and I44 of SSP, where I5 located in the N-terminal myristoylation motif and I31, I43, and I44 located in two helixes. The degree of variation of these amino acids varied among lineages. Taking I5 as an example, it underwent mutations in lineage II-VII, and the variation rates were all above 50%. Mutational analysis of GP1 showed that only S90N\T substitutions happened in N-glycosylation region, while asparagine replaced serine as the main amino acid in the lineage II-VII. It was worth noting that in lineage VI, threonine specifically appeared and was associated with strains isolated from humans. For GP2, the substitution of amino acids primarily appeared in N-terminal fusion peptide, heptad repeat and so on. The changes of GPC among lineages demonstrated the sequence diversity of LASV, and these changes mainly occurred in functional regions related to membrane fusion, which was highly likely to affect the regulation of related functions [36, 37].

**Fig. 7:**
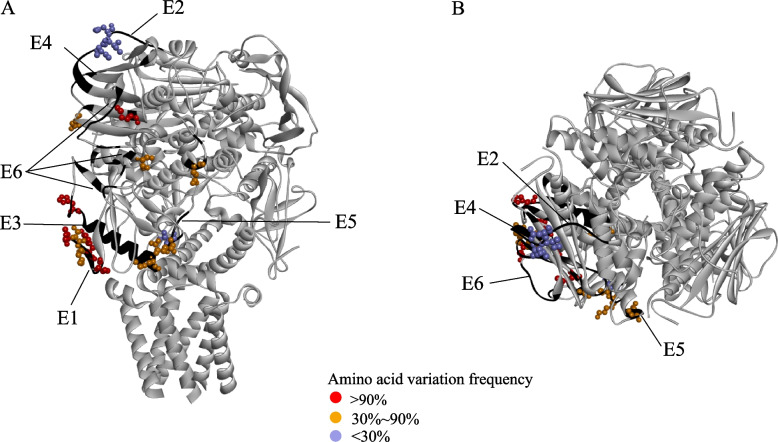
Global amino acid variation in epitopes mapped onto the protein crystal structure of the GPC trimer. Epitopes were plotted in the 7puy model, and each epitope is represented in black and marked in the figure. The variation sites on epitopes used different colours to represent the variation rate. See the description in the figure for the specific change rate

We also found amino acid substitutions in the known epitopes (Table 2). E1 (epitope 1) was reported to be the best predicted LBL epitope in GPC [38], but in our results, more than half of the amino acids in E1 showed changes among lineages. Spatial structure analysis showed E1 and E2 were located on the GPC trimer's surface, while E3 was present in heptad repeat 1 and the membrane proximal external region in GP2 (Fig. 7). E2 and E4 were located on the surface of GPC, and they both contained I112 and N114, indicating that they had a chance to bind with antibodies. E5 and E6 located in the membrane fusion region [39, 40] and antibody binding region [41], respectively. When changes occurred, the related functions were highly likely to be affected. These variations in amino acid mainly happened in lineage II, which led to the development and application of broad-spectrum vaccines.

**Table 2:**
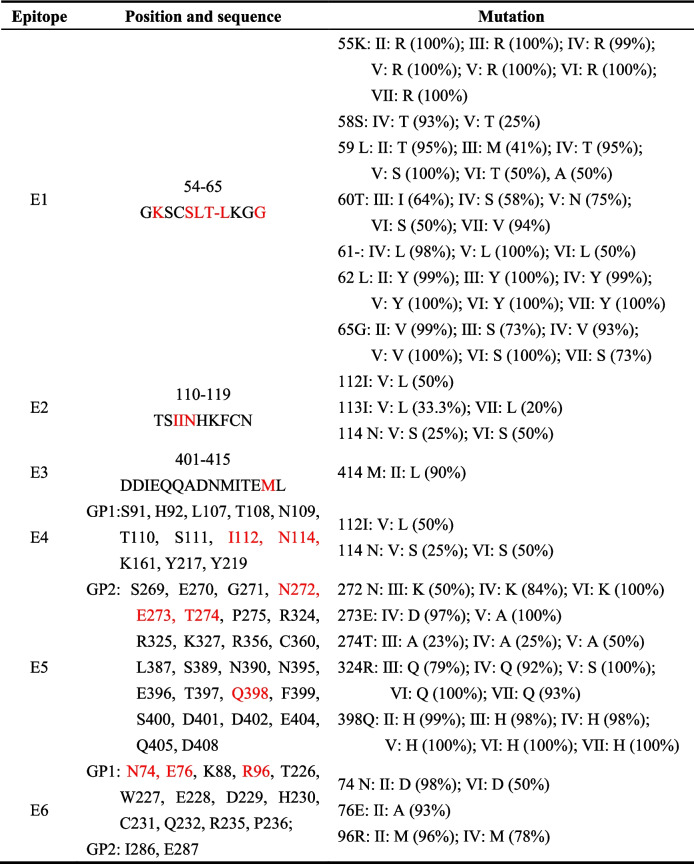
Amino acid variations with known epitopes

The epitope was the Josiah strain, but in our results, the amino acid at the mutation site was artificially replaced by the amino acid of the Pinneo strain to unify the mutation results. The red highlighted amino acid indicates the mutated amino acid

## Discussion

Spatial temporal dynamics analysis showed LASV had two transmission routes in West Africa, and the reasons for these seem to be influenced by natural reservoir spillovers and certain human activities. The natural host of LASV was mainly the *Mastomys natalensis*, which was semi-commensal habit with humans and had the characteristic of seasonal reproduction. A previous study showed that *Mastomys natalensis* reached its breeding peak at the end of the rainy season in West Africa [42]. When the rodent density increased, the ratio of their activity frequency to the crowd concentration area in the dry season also increased accordingly. This seasonal activity increased the probability of human infection, causing concentrated outbreaks of LASV in the dry season [2]. Moreover, the Triangular Trade route in the sixteenth century approximated the special and temporal traits of transmission route 2 and the armed conflicts in recent years also played an important role in the spatial distribution of LASV [43]. These human activities facilitated the incidental transport of rodents over geographical barriers, which may have been one of the reasons for the widespread spread of LASV in West Africa. Michael R once proposed that Liberia was the entry point of LASV into the MRU [11]. In route 2, the outbreak of the epidemic in West African countries was more closely linked to Liberia. Nigeria, as the source epicentre of LASV, spread the virus outward to Liberia, and then independently spread from Liberia to surrounding countries, resulting in a wider spread of LASV in West Africa. This implied Liberia may be a secondary epicentre for the outbreak of LASV on the West Africa.

Not all the family of *Arenaviridae* contained a bi-segmented genome, and recently, the new virus with tri-segmented genomes had been detected [44], indicating the generation of new segments may have been through long-term evolution [45]. This may also be one of the reasons for the inconsistent root TMRCA of the two segments of LASV. From 1969 to 2018, several heavy rainfall events occurred in West Africa, especially in Nigeria in 1988. The increase in rainfall affected the host's habits [43], thereby affecting the spread of LASV. The increase in genetic diversity during this period may be related to the cumulative effect of rainfall [46]. The virus lacking ZP was found in family of *Arenaviridae*, indicating that arenaviruses may have evolved a new mechanism that could replace ZP [44]. The high evolution rate of ZP in LASV suggested that LASV may also be undergoing similar evolution. And other study had shown that the high evolution rate may be related to its short length [47]. Thus, when considering ZP as a drug target [48], it was necessary to consider the potential risks.

Two reported strains [12] indicated that there had been reassorted within the sub-lineage IIg of LASV, and more reassortment signals further suggested that reassortment had been still ongoing. In the whole genome sequences obtained in Côte d'Ivoire, four potential reassortment strains were identified. Further investigations revealed that the occurrence of reassortment signal of LASV_3625 may be attributed to recombination events within L segment. For the L segment, four strains isolated from Côte d'Ivoire also showed significant recombination signals. The major and minor parents of these strains come from the lineage IV and V, respectively, indicating that these strains were produced by of recombination of two lineages. The strains of the Côte d'Ivoire branch were highly likely to be affected by recombination, thereby exacerbating the expansion of lineage V.

The diversity of LASV could be reflected through GPC, which served as a surface antigen and often was used as a candidate immunogen in vaccine development [35]. Multiple residues substitutions also emerged in GPC. At E178, each lineage contained at least two mutated amino acids, and the continuous substitutions of amino acids was likely related to their positive selection or high Shannon entropy of GPC [49]. Analysis of known epitopes showed that they were also undergoing amino acid substitutions. These changes in E2 and E4 sequences were mainly concentrated in lineages V to VII, while they were relatively conserved in the I-IV lineages. This special variation among lineages may be beneficial for them as candidate epitopes for specific vaccines in lineages I to IV.

## Conclusions

The results of this study investigated the temporal and spatial dynamics of LASV transmission in West Africa and its associated factors. We have highlighted the crucial role of Liberia as a secondary epicenter and estimated relevant evolutionary features such as TMRCA, effective population size, and evolution rate. Relevant results suggested that multiple lineages contained reassortment events, especially the sub-lineage IIg, while recombination played a role in expanding lineage V. Notably, significant amino acid variations in GPC demonstrated the sequences diversity of LASV, and this diversity change may affect the normal expression of epitopes.

### Supplementary Information

**Supplementary Material 1.**

**Supplementary Material 2.**

**Supplementary Material 3.**

**Supplementary Material 4.**

**Supplementary Material 5.**

## Data Availability

All the relevant datasets relating to this study are available upon reasonable request to the co-authors.

## Abbreviations

LF: Lassa fever
LASV: Lassa virus
MRU: Mano River Union
L: Large
LP: RNA-dependent RNA polymerase
ZP: Zinc-binding protein
S: Small
GPC: Glycoprotein precursor
NP: Nucleoprotein
SSP: Stable signal peptide
NCBI: National Center for Biotechnology Information
ML: Maximum likelihood
MCC: Maximum clade credibility
LBL: Linear B-cell lymphocyte
TMRCA: The time of the most recent common ancestor
E1: Epitope 1
