# Supplementary material for: Spatio-temporal spread and evolution of Lassa virus in West Africa
Source: BMC Infect Dis. 2024 Mar 14;24:314. doi: 10.1186/s12879-024-09200-8 (PMC10941413; doi:10.1186/s12879-024-09200-8)
Supplement: Supplementary file 3 — Supplementary Material 3. [file 12879_2024_9200_MOESM3_ESM.zip › Figure S1_S.pdf]

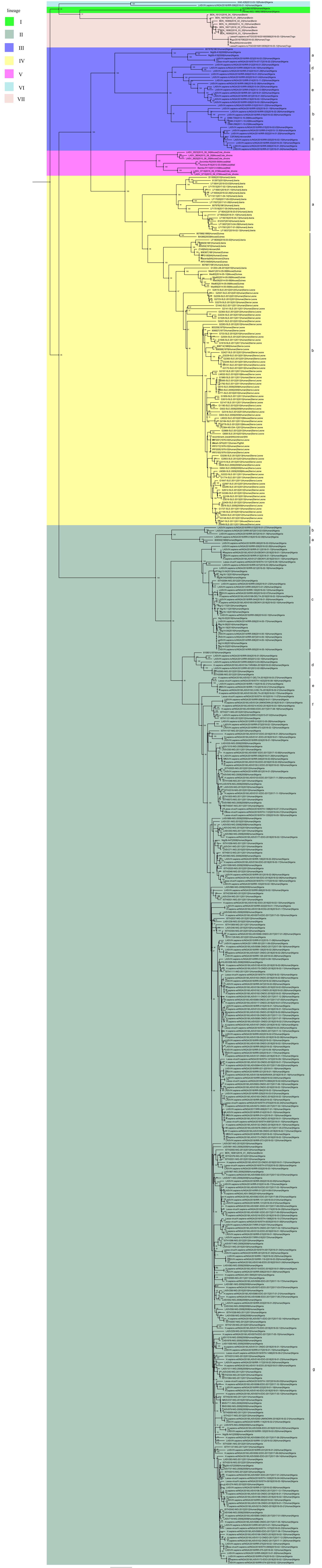

Figure S1 ML tree of S segment nucleotide sequences. Each colour represented a different lineage, where the posterior probability of each node was represented by numbers, and each sub-lineage was individually labeled with letters.
