# Supplementary figures and images for: Spatio-temporal spread and evolution of Lassa virus in West Africa
Source: BMC Infect Dis. 2024 Mar 14;24:314. doi: 10.1186/s12879-024-09200-8 (PMC10941413; doi:10.1186/s12879-024-09200-8)

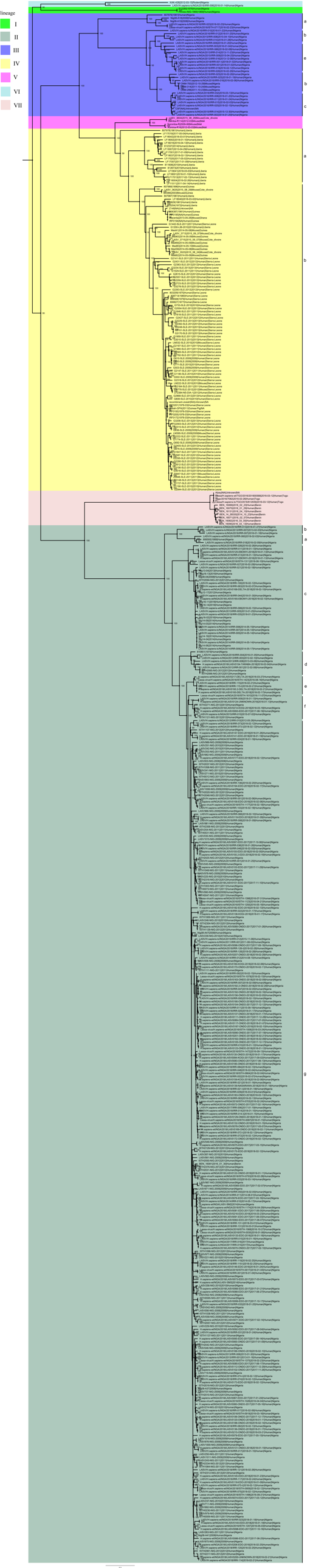

Supplement: Supplementary file 3 — Supplementary Material 3. [file 12879_2024_9200_MOESM3_ESM.zip › Figure S1_L.pdf]
