# Supplementary material for: Spatio-temporal spread and evolution of Lassa virus in West Africa
Source: BMC Infect Dis. 2024 Mar 14;24:314. doi: 10.1186/s12879-024-09200-8 (PMC10941413; doi:10.1186/s12879-024-09200-8)
Supplement: Supplementary file 4 — Supplementary Material 4. [file 12879_2024_9200_MOESM4_ESM.pdf]

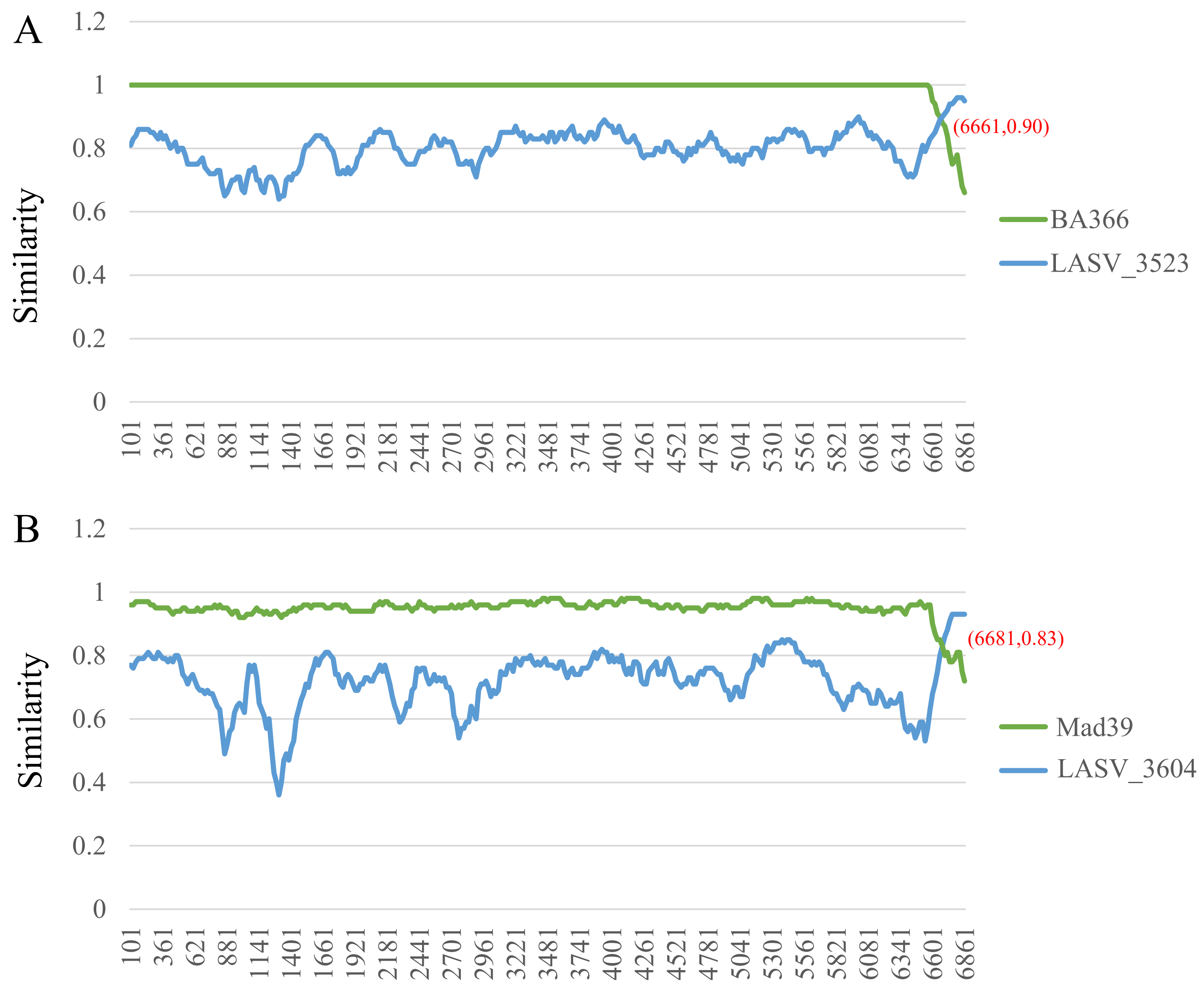

Figure S2 Similarity between recombined strains and their parents. The strains LASV_3713 (A) and LASV_3523(B) show their own similarity with their parents. The major parent and minor parent are represented by the green line and green blue line, respectively. The red coordinate points represent the breakpoint position and the similarity.
