# Supplementary material for: Spatio-temporal spread and evolution of Lassa virus in West Africa
Source: BMC Infect Dis. 2024 Mar 14;24:314. doi: 10.1186/s12879-024-09200-8 (PMC10941413; doi:10.1186/s12879-024-09200-8)
Supplement: Supplementary file 5 — Supplementary Material 5. [file 12879_2024_9200_MOESM5_ESM.docx]

**Table S1** TMRCA of each lineage of LASV

| **Major lineage** | **Sub-lineage** | **L segment** | | | **S segment** | | |
| --- | --- | --- | --- | --- | --- | --- | --- |
|  |  | **median** | **95%HPD** | **median** | | **95%HPD** |  |
| root |  | 634 | [385-879] | 1224 | | [1030-1401] |  |
| Ⅰ |  | \ | \ | \ | | \ |  |
| Ⅱ |  | 1666 | [1607-1730] | 1720 | | [1348-1785] |  |
|  | Ⅱa | 1936 | [1922-1950] | 1935 | | [1915-1953] |  |
|  | Ⅱb | \ | \ | \ | | \ |  |
|  | Ⅱc | 1968 | [1900-1939] | 1928 | | [1906-1949] |  |
|  | Ⅱd | 1932 | [1912-1940] | 1930 | | [1912-1942] |  |
|  | Ⅱe | \ | \ | \ | | \ |  |
|  | Ⅱf | 1928 | [1912-1944] | 1933 | | [1912-1952] |  |
|  | Ⅱg | 1941 | [1928-1953] | 1943 | | [1912-1946] |  |
| Ⅲ |  | 1319 | [1197-1446] |  | |  |  |
|  | Ⅲa | 1816 | [1781-1852] | 1825 | | [1778-1866] |  |
|  | Ⅲb | 1937 | [1920-1953] | 1937 | | [1914-1957] |  |
|  | Ⅲc | 1972 | [1962-1981] | 1976 | | [1965,1987] |  |
|  | Ⅲd | \ | \ | \ | | \ |  |
|  | Ⅲe | 1828 | [1793-1862] | 1833 | | [1787,1876] |  |
| Ⅳ |  | 1572 | [1493-1647] | 1703 | | [1630-1719] |  |
|  | Ⅳa | 1830 | [1807-1863] | 1845 | | [1805,1882] |  |
|  | Ⅳb | 1747 | [1703-1792] | 1731 | | [1665,1791] |  |
| Ⅴ |  | 1854 | [1821-1886] | 1839 | | [1794-1880] |  |
| Ⅵ |  | 1430 | [1307,1546] | 1625 | | [1513-1713] |  |
| Ⅶ |  | 1958 | [1946-1969] | 1964 | | [1954-1978] |  |

\: This lineage and sub-lineage only contain one strain, so no relevant results are calculated.
